# Retraction: The ING4 Binding with p53 and Induced p53 Acetylation were Attenuated by Human Papillomavirus 16 E6

**DOI:** 10.1371/journal.pone.0331481

**Published:** 2025-09-03

**Authors:** The PLOS One Editors

After this article [1] was published, concerns were raised about Figs 1 and 2.

Specifically, when levels are adjusted to visualize the background:

- The following lanes appear similar:
  - In Fig 1A, lanes 2-3 of the E6 - IP: E6 panel and lanes 2-3 of the ING4 - IP: E6 panel.
  - In Fig 1B, lanes 2 and 4 of the ING4 panel.
  - In Fig 1B, lane 3 of the ING4 panel, and in Fig 1C, lane 2 of the E6 panel, when flipped horizontally.
  - In Fig 1B, lanes 2 and 4 of the ING4 panel, and in Fig 1C, lane 3 of the E6 panel, when flipped horizontally.
  - In Fig 1D, lanes 3 and 6 of the E6 panel.
  - In Fig 2D, the Caski shE6 and CasKi shC panels and Fig 2A of [2], despite representing different cell transfections.
- There appear to be vertical discontinuities in the following panels:
  - In Fig 1B, in the E6 and ING4 panels.
  - In Fig 1C, in the E6 panel.
  - In Fig 1D, in the E6 panel.
- There appear to be multiple regions of similarity, and/or regions which are discontinuous with adjacent areas, in the backgrounds of the following panels:
  - In Fig 1C, in lanes 2 and 3 of the Coomassie panel.
  - In Fig 1D, in lane 2 of the ING4 panel.
  - In Fig 2B, in lane 5 of the ING4 panel.
  - In Fig 2B, in lane 5 of the Flag-E6 panel.

The authors did not respond to PLOS’ requests for comment or original data underlying the figures of concern.

In light of the above unresolved concerns, the *PLOS One* Editors retract this article.

All authors either did not respond directly or could not be reached.
